# Contribution of the Japan International Cooperation Agency health-related projects to health system strengthening

**DOI:** 10.1186/1472-698X-13-39

**Published:** 2013-09-22

**Authors:** Motoyuki Yuasa, Yoshie Yamaguchi, Mihoko Imada

**Affiliations:** 1 Department of Public Health, Juntendo University Graduate School of Medicine, Hongo 2-1-1 Bunkyo-ku, Tokyo 113-8421, Japan; 2 Graduate School of Medical Administration, Tokyo Medical and Dental University, Yushima 1-5-45 Bunkyo-ku, Tokyo 113-8510, Japan; 3 Department of Tropical Medicine and Parasite Infection, School of Medicine, Keio University, Shinano-machi 35, Shinjuku-ku, Tokyo 160-8582, Japan

**Keywords:** Japan International Cooperation Agency (JICA), Health system improvement, Project design matrix

## Abstract

**Background:**

The Japan International Cooperation Agency (JICA) has focused its attention on appraising health development assistance projects and redirecting efforts towards health system strengthening. This study aimed to describe the type of project and targets of interest, and assess the contribution of JICA health-related projects to strengthening health systems worldwide.

**Methods:**

We collected a web-based Project Design Matrix (PDM) of 105 JICA projects implemented between January 2005 and December 2009. We developed an analytical matrix based on the World Health Organization (WHO) health system framework to examine the PDM data and thereby assess the projects’ contributions to health system strengthening.

**Results:**

The majority of JICA projects had prioritized workforce development, and improvements in governance and service delivery. Conversely, there was little assistance for finance or medical product development. The vast majority (87.6%) of JICA projects addressed public health issues, for example programs to improve maternal and child health, and the prevention and treatment of infectious diseases such as AIDS, tuberculosis and malaria. Nearly 90% of JICA technical healthcare assistance directly focused on improving governance as the most critical means of accomplishing its goals.

**Conclusions:**

Our study confirmed that JICA projects met the goals of bilateral cooperation by developing workforce capacity and governance. Nevertheless, our findings suggest that JICA assistance could be used to support financial aspects of healthcare systems, which is an area of increasing concern. We also showed that the analytical matrix methodology is an effective means of examining the component of health system strengthening to which the activity and output of a project contributes. This may help policy makers and practitioners focus future projects on priority areas.

## Background

Resources devoted to global healthcare systems are disproportionately allocated and not commensurate with the distribution of health problems. Low- and middle-income countries account for 11% of global health expenditure, but 93% of the world’s health burden is borne by 84% of the population living in these countries [1]. To reduce healthcare gaps, many practitioners and policy makers in healthcare development have focused on identifying the most effective methods of improving health service provisions [2]. In the early 1990s, multilateral donor agencies, such as the World Bank, launched health sector reform projects in many developing countries [3,4]. Since 2000, in particular, a large number of donor agencies, international organizations and non-governmental organizations have recognized the importance of health system strengthening in promoting sustainable, autonomous progress toward achieving the Millennium Development Goals [5]. The World Health Organization (WHO) defines health system strengthening as a process of identifying and implementing changes in policy and practice to improve one or more of the functions of the system to respond better to challenges [6]. Programs targeting specific diseases, treatments or preventative strategies, such as the Global Alliance on Vaccines Initiative (GAVI), Global Fund to fight AIDS, Tuberculosis and Malaria (GFATM) and Treat, Train and Retain Initiative (TTR) have included a funding component for health system strengthening [7,8].

In order to understand the structure, function and performance of a health system, many researchers have developed health system models with interactive components and have assessed performance using various indicators [9-11]. The WHO developed a health system framework and rated health system performance of its member states in 2000 [1]. Subsequently, the WHO revised its health system framework to include six building blocks, specifically: service delivery; health workforce (‘workforce’); information; medical products, vaccines and technologies (‘medical products’); financing; and leadership/governance (‘governance’) [12]. Recently, there has been a growing recognition of the need to analyze health system performance using this framework [13-15]. This conceptual framework has been widely used in outlining an entire health system and in analyzing the contribution of interventions to strengthening health systems [16].

In the global context, the Japan International Cooperation Agency (JICA) has focused its attention on appraising health development assistance projects and redirecting efforts toward strengthening health systems. The JICA has been in recent years one of the largest bilateral development organizations with a network of about one hundred oversee offices, projects in about 150 countries, and available financial resources of approximately 9.6 billion US dollars in 2010. However, although on average approximately 40 technical health-related projects have been implemented annually anywhere in the world, so far these projects have seldom been evaluated systematically from a health systems perspective. Accordingly, we aimed to describe the types of JICA health-related projects and targets of interest by examining their impact on each of the WHO’s building blocks. In particular, we assessed the contribution of the JICA projects to health system strengthening. To accomplish this, we developed a method by which we could examine the configuration of different healthcare systems. Since 2007, most health system appraisals have been undertaken with reference to the WHO’s framework of six building blocks, but the JICA has designed its projects using a framework known as the Project Design Matrix (PDM). We developed an analytical matrix of program activity and output in which the WHO’s framework and the PDM have been integrated in order to assess JICA’s projects from a view of the WHO building blocks.

## Methods

### Data sources for JICA projects

**Figure 1:**
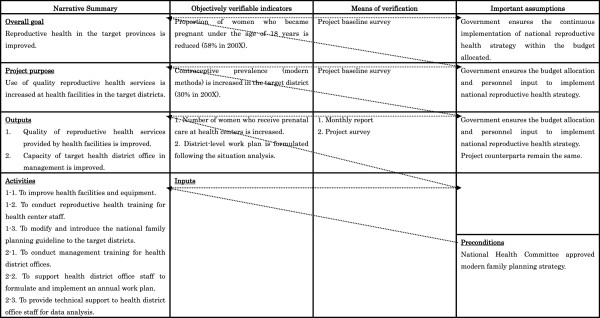
Example of Project Design Matrix of a Japan International Cooperation Agency project.

Generally, JICA technical cooperation projects are designed following a logical framework: a 16-cell matrix with four columns and four rows known as the PDM [17]. It serves as a management tool for efficiently designing, monitoring and evaluating a project at every level, has been widely used by bilateral and multilateral donor organizations, and is employed in a participatory process of project design (Project Cycle Management). The Japanese Foundation for Advanced Studies on International Development (FASID) adopted and modified the PDM framework as its primary project design and management tool for JICA programs. The JICA project manager and practitioner are required to monitor and evaluate progress using the PDM. The PDM summarizes the narrative of activities, outputs, project purpose and overall goal of the relevant project [18]. In general, as arrow shows the process in Figure 1, projects proceed in the following stages; precondition, activity, assumption, outputs, assumption, project purpose, assumption, overall goal. The project is launched when preconditions exist, and then activities are initiated. An output may comprise several activities; accordingly, a number of activities can be nested within one output. In turn, multiple integrated outputs may comprise a project’s purpose. Put simply, outputs are produced through activities, while the project purpose is achieved through these outputs. The PDM also establishes a link between the narrative summary and financing and resource inputs, as well as assumptions that may critically influence the progress of the project. Thus, reviewing the PDM can readily facilitate an understanding of the configuration and characteristics of a given project. Figure 1 shows a partially modified and simplified example of the PDM in which the narrative summary of the project for improving reproductive health in Syria is presented.

From the JICA website home page, we collected the PDMs of JICA technical health-related projects conducted worldwide between January 1, 2005 and December 31, 2009 as well as those that were ongoing. This collection of PDMs over 5 years provided an adequate sample of more than 100 projects. The PDMs obtained were publicly available on the JICA website (URL: http://gwweb.jica.go.jp/km/ProjectView.nsf/VW02040105?OpenView&Start = 1&Count = 1000&Expand = 2#2, accessed June 15, 2013, available only in Japanese). One hundred forty-eight PDMs were available, of which 105 were finally analyzed. The study excluded 20 PDMs for programs concerning training courses, 16 with incomplete or missing outputs and activities, and seven PDMs pertaining to a follow-up program.

### Analytical matrix and classification criteria

From a health system perspective, all components of the PDM outputs were categorized by two researchers (YY and MI) into one of the six building blocks that comprise the WHO framework. Each output consisted of one or more activities, and each activity in the given output was also classified into one of the six building blocks by the same researchers. This resulted in a fractal configuration that included activities in six blocks nested within outputs that had also been categorized into six blocks.

**Figure 2:**
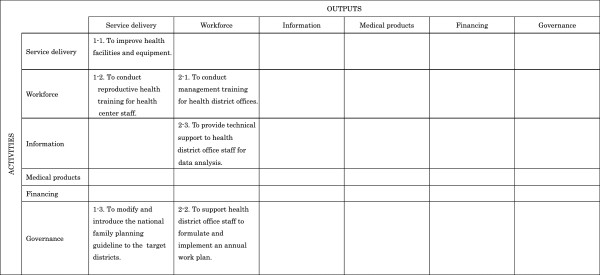
Example of analytical matrix of outputs and activities.

In order to analyze the contribution of each JICA project to health system strengthening, we developed an analytical matrix. This matrix was formed by the row and column of the six blocks corresponding to the outputs and activities in each PDM, respectively. Figure 2 shows an example of the analytical matrix to which the PDM in Figure 1 was applied. As the first column on the horizontal axis of the analytical matrix describes output, and the output of the example project was classified as SD, this example was classified as a type of Service Delivery (SD) plus Workforce. and therefore is grouped under SD The analytical algorithm for outputs and activities followed the classification criteria shown in Table 1[12]. A third researcher (MY) checked the categorized data; any data determined to have been misclassified were re-categorized based on the consensus of the three researchers.

**Table 1:** Classification criteria by six building blocks

																			**Building blocks**																																																												**Contents**
																			Service delivery																																																												• Provision of service package
																																																																															• Strengthening of service delivery system
																																																																															• Promotion of awareness of service demand
																																																																															• Public health education targeting
																																																																															• Capacity development of health volunteers
																			Health workforce																																																												• Strengthening of supply system of health workforce
																																																																															• Capacity development of health workforce (training, workshops, et cetera)
																																																																															• Supervision
																			Information																																																												• Production, collection, analysis and use of health related information and data (monitoring and evaluation)
																																																																															• Development of health educational materials
																																																																															• Dissemination and advocacy of the results (homepage on web, newsletter, seminar, et cetera)
																																																																															• Conduct of health research
																			Medical products, vaccine and technologies																																																												• Medical products
																																																																															• Vaccines
																																																																															• Appropriate technologies
																																																																															• Strengthening of logistic supply system
																			Financing																																																												• Prepayment
																																																																															• Collection of finances
																																																																															• Pooling of finances
																																																																															• Purchasing of resources
																			Leadership and governance																																																												• Capacity development for decision making, legislation and regulation
																																																																															• Capacity development for administration, oversight and guidance
																																																																															• Strengthening of organizational and institutional management
																																																																															• Establishment of monitoring and evaluation system
																																																																															• Establishment of networks

### Analysis of the JICA projects

First, in order to understand the overall characteristics of the JICA technical health-related projects, we classified the projects into types according to the combination of the outputs. The type was identified by a building block designated by the main output. For example, a project having a main output of service delivery (SD) was categorized as an SD type. SD projects generating additional outputs, such as workforce, information or medical products were classified as SD plus workforce, SD plus information, and SD plus medical products, respectively, regardless of inclusion of governance output. The SD was found to be far more frequently the main output compared to the other blocks of the WHO framework according to their classification, and therefore, it was treated differently in the presentation of the findings.

Projects having main outputs of workforce, information, medical products, and financing, as well as those with more than three outputs were categorized as workforce, information, medical products, financing and mixed type, respectively, regardless of the presence of governance. Projects consisting of a governance output alone were categorized as such. In our study, since governance was regarded as an overarching component to manage other blocks and generate quality health service, it was considered to be an independent block.

Second, to examine the health fields (specific health issues or programs) to which the JICA projects contributed, we identified the main target field of the projects based on the primary purpose of the PDMs and the project titles. The field ‘health system’ (HS) was defined as a project aimed primarily at developing the capacity of health professionals and maintaining medical equipment at health institutes, without regard to a specific health problem or disease. Maternal and child health (MCH) included improvement of child health services and child nutrition.

Third, we tested for associations between project types and health fields using a standardized method for correspondence analysis of categorical data (SPSS statistics version 21, IBM Japan, Tokyo, Japan) [19]. By displaying the rows (types) and columns (health fields) in a two-way contingency table, correspondence analysis presents a geometrical association, indicating the extent to which a health field is likely related to a type.

Finally, the number of activities comprising each PDM output was counted in each cell of the analytical matrix in order to investigate the frequency of outputs and activities, and to identify the JICA’s focus of interest.

## Results

Table 2 shows the duration of implementation of each of the JICA projects by a targeted country. The most frequent duration of the projects was 3 years, followed by 4-year periods (range 10 months – 7 years). There were notable differences in the number of projects undertaken per country, ranging from one to six in Zambia and China, with most projects being based in Asia.

**Table 2:** Target country and duration of the Japan International Cooperation Agency projects between 2005 and 2009

**Region / country**		**Duration of project**							**No.**
		**< 1 year**	**1 year**	**2 years**	**3 years**	**4 years**	**5 years**	**7 years**	**Total (%)**
Africa									39 (37)
	Burundi	0	0	0	1	0	0	0	1
	Egypt	0	0	2	0	1	0	0	3
	Eritrea	0	0	0	1	0	0	0	1
	Ethiopia	0	0	0	0	0	2	0	2
	Ghana	1	0	0	0	2	0	0	3
	Kenya	0	0	0	4	0	0	0	4
	Madagascar	0	0	0	1	1	0	0	2
	Malawi	0	0	0	0	1	0	0	1
	Mozambique	0	0	0	3	0	0	0	3
	Niger	0	0	0	1	0	0	0	1
	Nigeria	0	0	0	0	1	0	0	1
	Senegal	0	0	1	1	0	0	0	2
	Sierra Leone	0	0	0	1	0	0	0	1
	South Africa	0	0	0	1	1	0	0	2
	Sudan	0	0	0	2	0	0	0	2
	Tanzania	0	0	0	1	1	0	0	2
	Uganda	0	0	0	1	0	0	0	1
	Zambia	0	0	2	2	1	1	0	6
	Zimbabwe	0	0	0	1	0	0	0	1
Asia									43 (41)
	Afghanistan	0	0	1	2	0	1	0	4
	Bangladesh	0	0	0	0	1	0	0	1
	Bhutan	0	0	0	1	0	0	0	1
	Cambodia	0	0	0	3	0	0	0	3
	China	0	0	1	4	0	1	0	6
	India	0	0	0	0	1	0	0	1
	Indonesia	0	0	0	4	0	1	0	5
	Laos	0	0	0	2	2	1	0	5
	Myanmar	0	0	1	0	0	2	1	4
	Nepal	0	0	0	0	1	0	0	1
	Pakistan	0	0	0	2	0	0	0	2
	Philippines	0	0	0	0	1	1	0	2
	Sri Lanka	0	0	0	1	0	0	0	1
	Thailand	0	0	0	1	1	0	0	2
	Viet Nam	0	0	0	2	2	1	0	5
Latin America									15 (14)
	Bolivia	0	0	0	0	1	1	0	2
	Brazil	0	0	0	0	0	1	0	1
	Chile	0	0	0	1	0	0	0	1
	El Salvador	0	0	2	0	0	0	0	2
	Guatemala	0	0	0	1	1	0	0	2
	Honduras	0	0	0	1	1	0	0	2
	Nicaragua	0	0	0	0	1	1	0	2
	Paraguay	0	0	0	2	0	0	0	2
	Peru	0	0	0	0	1	0	0	1
Middle East									4 (4)
	Iraq	0	1	0	0	0	0	0	1
	Palestine	0	0	0	1	0	0	0	1
	Syria	0	0	0	1	0	0	0	1
	Yemen	0	0	0	0	1	0	0	1
Oceania									4 (4)
	Fiji	0	0	1	1	0	0	0	2
	Pacific States	0	0	0	0	0	1	0	1
	Solomon	0	0	0	1	0	0	0	1
Total		1	1	11	52	24	15	1	105 (100)

Table 3 presents the 105 JICA health-related projects categorized by types and health field. The row for SD in the table provides a subtotal for each project subtype, including SD alone, SD plus workforce, SD plus information and SD plus products. The most frequent project type was SD (n = 45), followed by workforce (n = 34). Since the SD included 21 workforce outputs and all the mixed types involved workforce outputs, more than half JICA projects (59.0%, 62 out of 105) had contributed to workforce. Few projects concentrated exclusively on information, medical products, or governance. Only two projects included an output for financing: a project in the Philippines that aimed at holistic health system strengthening and a mixed type project in Pakistan that aimed to establish an information system. The most frequent health field was health system (HS, n = 46), followed by the prevention of AIDS, tuberculosis and malaria (ATM, n = 20), and maternal and child health (MCH, n = 13). Projects targeting prevention and control of infectious diseases other than ATM (ID), the Expanded Program of Immunization (EPI) and Blood Transfusion against HIV infection (BT) ranked second among the health fields (n = 33).

**Table 3:** Type of Project Design Matrix outcomes for Japan International Cooperation Agency health-related projects by health field

**Type of PDM output**	**Total**	HS^*^	**MCH†**	**RH**	**ATM**	**Infectious diseases other than ATM**	**EPI**	**SH**	**FS**	**MH**	**BT**
Service delivery (SD)	45	8	10	7	13	2	2	2	0	0	1
SD alone	17	5	5	1	4	0	1	0	0	0	1
SD+Workforce	21	2	5	6	6	0	0	2	0	0	0
SD+Information	4	0	0	0	2	2	0	0	0	0	0
SD+Products	3	1	0	0	1	0	1	0	0	0	0
Workforce	34	22	3	1	2	2	1	0	2	1	0
Information	8	4	0	0	3	1	0	0	0	0	0
Medical Products	4	0	0	0	1	1	2	0	0	0	0
Financing	1	1	0	0	0	0	0	0	0	0	0
Mixed‡	7	6	0	0	1	0	0	0	0	0	0
Governance alone	6	5	0	0	0	1	0	0	0	0	0
Total (%)	105 (100)	46 (43.8)	13 (12.4)	8 (7.6)	20 (19.0)	7 (6.7)	5 (4.8)	2 (1.9)	2 (1.9)	1 (1.0)	1 (1.0)

*Abbreviations PDM* Project Design Matrix, *HS* health system, *MCH* maternal and child health, *RH* reproductive health, *ATM* prevention of *AIDS* tuberculosis and malaria, *EPI* expanded program on immunization, *SH* school health, *FS* food safety, *MH* mental health, *BT* blood transfusion.

*Health system includes capacity development, maintenance of medical equipment, and traditional medicine.

†Maternal and child health includes improvement of nutrition.

‡Mixed includes various outputs of the PDM.

**Figure 3:**
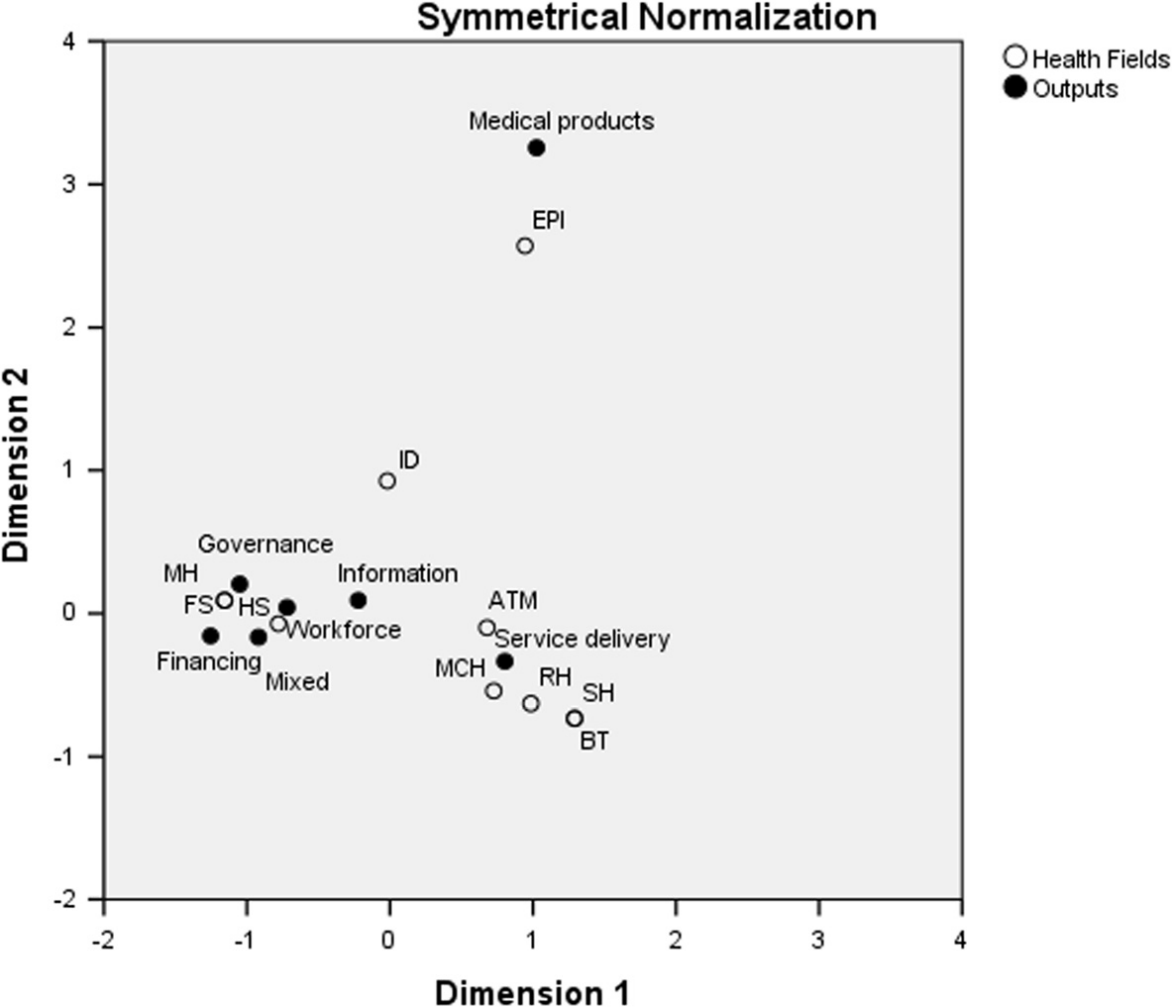
**Association between outputs and health fields by correspondence analysis.** Abbreviations: HS, health system; MCH, maternal and child health; RH, reproductive health; ATM, prevention of AIDS, tuberculosis and malaria; ID, Infectious diseases other than ATM; EPI, expanded program on immunization; SH, school health; FS, food safety; MH, mental health, BT, blood transfusion.

Figure 3 illustrates the association between project types and health fields as determined by correspondence analysis. The total inertia (total variance explained) was 0.694, indicating that in our model, the project types explained around 69.4% of the variation within the health fields. The mutual distances between medical products and EPI, service delivery and ATM, MCH and reproductive health (RH), and workforce and HS were very small, meaning that the EPI projects contributed substantially to the provision of medical products, vaccines and technologies. The projects concerning vertical targets such as ATM, MCH and RH tended to focus on enhancing service delivery, whereas projects for holistic health system strengthening tended to engage in workforce capacity development.

Table 4 shows the distribution of activities in the PDMs by output. The JICA projects were highly likely to contribute to enhancing the blocks for governance (total number of activities, n = 257), workforce (n = 158), and service delivery (n = 134) via outputs, while at the activity level, the projects were inclined to contribute to enhancing the blocks for governance (n = 200), workforce (n = 169), and information (n = 133). In particular, the findings suggested that the outputs of workforce and governance mostly included workforce activities. JICA projects focused on ‘soft’ assistance such as the workforce and governance building blocks, whereas ‘hard’ support for finance and medical products was not evident in the respective outputs and activities.

**Table 4:** Number of activities by type of Project Design Matrix outputs in Japan International Cooperation Agency projects

**Activities**	**Outputs**						
	**Service delivery**	**Workforce**	**Information**	**Medical products**	**Financing**	**Governance**	**Total**
**Service delivery**	30	16	0	0	1	23	70
**Workforce**	31	58	12	3	0	65	169
**Information**	27	28	13	3	0	62	133
**Medical products**	7	5	2	5	0	14	33
**Financing**	0	1	0	0	0	2	3
**Governance**	39	50	14	4	2	91	200
**Total**	134	158	41	15	3	257	608

## Discussion

We analyzed the configuration of PDMs for JICA health-related projects using an analytical matrix with a link between the project PDM and the WHO’s conceptual health system framework. This study revealed the common characteristics of the JICA projects for technical assistance in health. The majority of JICA projects had prioritized assistance such as enhancement of the workforce and governance, as well as improvement of service delivery. Conversely, support for medical products and financing was modest. These findings reflect the JICA consensus statement, which emphasizes bilateral cooperation via capacity development. In particular, the JICA stresses the role of the health field in strengthening the health system, preventing and controlling infectious diseases including ATM and others, and MCH, which together accounted for 87.6% of JICA assistance. When examined in detail from the perspective of the health system, JICA projects appeared to be targeted at achieving governance, workforce and service delivery outputs through investment in activities involving the governance, workforce and information blocks. In particular, nearly 90% of the JICA technical assistance in health had directly focused on improving governance as the most crucial tactic for accomplishing its objectives.

Adam et al [13] reported that most health policy and system research publications have focused on human resources, service delivery and governance in low- and middle-income countries. Likewise, our results showed that the majority of JICA projects included in our analysis had contributed toward improving workforce capability and leadership, as well as governance. This implies that strengthening human resources and management functions were absolutely essential for achieving the goals of the projects [20]. Since sustainability and autonomy are important for healthcare development, the enhancement of perpetual and autonomous management capability among the workforce is a crucial component of project activities [21]. This underpins the rationale for JICA support for projects that strengthen management and governance through professional training and the creation of health networks [22,23]. Additionally, the JICA has provided many opportunities for capacity development by conducting training courses offering support extending beyond project-type assistance [24].

Finance function is important to strengthening the health system in every nation [25-27]. Nonetheless, the majority of the JICA projects did not include activities for improving financial systems. This may simply be a consequence of lack of financial expertise among the JICA experts. While recruiting Japanese experts to participate in health-related projects, it is a common practice of the JICA to seek specialists in health and medical sciences from universities and educational and research institutes and organizations, rather than financial organizations. This restrictive recruitment process might have resulted in a paucity of finance specialists. In general, irrespective of the donor, interventions to finance organizations of recipient countries can be sensitive political matters [28,29]. Assistance to strengthen financing systems may require not only specialists in the field, but also that they are afforded the power to make discreet and effective interventions.

We found that nearly half the projects analyzed were classified as health system strengthening; however, these projects did not comprehensively contribute to multiple health system blocks [16]. Of 46 projects in the HS column in Table 3, workforce enhancement was the primary target for 22 and eight had focused on service delivery; only six were categorized as mixed type. These findings indicate that some projects that aimed to strengthen health systems had not always contributed to all of the building blocks. Instead, these projects focused on workforce development, service delivery and providing support to the governance block without any attention to financial aid. For example, a JICA project classified as a ‘governance only’ project that aimed to strengthen district health services in the Morogoro region of Tanzania had focused only on capacity development in the governance block within public health sector hierarchies, and on communication among vertical and horizontal health institutions. Also, a JICA project to strengthen the regional health network in Santa Cruz, Bolivia, had outputs and activities in the workforce and governance blocks to expand holistic assistance for improving the health network; however, this had been categorized as a ‘workforce’ project. Thus, many JICA projects classified as health system strengthening did not always provide input to all blocks, but instead had implemented assistance in alternative blocks such as workforce and governance.

The correspondence analyses revealed that the EPI project was associated with the medical products block. The vertical projects implemented for fields such as ATM, MCH and RH shared a close relationship with the service delivery block, while the projects for health system strengthening were also strongly related to workforce. These results match the EPI project’s primary aim to enhance procurement, storage and distribution of vaccines, consistent with categorization in the medical products block. Furthermore, vertical programs for ATM, MCH and RH mainly strove to provide universal high quality services relating to their fields. The close association between HS and workforce indicated that most of the projects under the title of health system strengthening aimed to improve workforce capability.

Our study had several limitations. Although collecting data from all available PDMs might have minimized selection bias, the process of categorization may have resulted in informational bias due to the difficulty in interpreting ambiguous and obscure descriptions contained in some of the PDMs. A second important limitation was related to confidentiality of the data source. One possible method for examining and systematically comparing the JICA projects would have been to use the PDM alone; however, there is a fundamental analytical limitation to employing the PDM in order to examine the project type, health field, characteristics and the association between outputs and activities of the project. The PDM does not necessarily include an exhaustive list of elements that the project entails, and it is quite usual for the PDM to be amended or modified during its implementation. The limited information from the PDM should be taken into account when interpreting the study findings.

## Conclusions

To the author’s knowledge, this study is the first to examine the type and targets of interest of the JICA projects from the comprehensive perspective of a health system. Nearly half the projects were devoted to health system strengthening, followed by the prevention and control of infectious diseases, and maternal and child health, which together accounted for almost 90% of the assistance. An overwhelming majority of the JICA projects had contributed to building blocks for workforce and governance, and, as a result, service delivery through both outputs and activities. In contrast, the assistance for medical products and financing was minimal. Recently, the global health society has been paying increasing attention to innovative international financing programs for health, protection from financial catastrophe and impoverishment, and universal health coverage [30,31]; the need for support for financial systems is gradually increasing. Our findings suggest that JICA should not focus only on workforce capacity development and governance, but also on strengthening the financial functioning of health systems.

Our study also demonstrated how an analytical matrix can be used to elucidate which block a health-related project contributes to as output and activity. This method can be used to search which block of the system project managers and practitioners need to address from a health system perspective, and this methodology may be applicable to programs and projects other than the JICA.

## Abbreviations

GAVI: Global Alliance on Vaccines Initiative; GFATM: Global Fund to fight AIDS, Tuberculosis and Malaria; TTR: Treat, Train and Retain Initiative; WHO: World Health Organization; JICA: Japan International Cooperation Agency; PDM: Project Design Matrix; FASID: Japanese Foundation for Advanced Studies on International Development; SD: Service delivery; HS: Health system; MCH: Maternal and child health; ATM: AIDS, tuberculosis and malaria; ID: Infectious diseases other than AIDS, tuberculosis or malaria; EPI: Expanded Program of Immunization; BT: Blood transfusion against HIV; RH: Reproductive health.

## Competing interests

We declare that we have no conflict of interests.

## Authors’ contributions

MY made substantial contributions to conception and design, analysis of data, and wrote the paper and was responsible for the final submission of the paper. YY and MI made contributions to analyses of data. All authors read and approved the final manuscript.

## Pre-publication history

The pre-publication history for this paper can be accessed here:

http://www.biomedcentral.com/1472-698X/13/39/prepub
